# Extract from the Records of the Atlanta Medical Society

**Published:** 1856-04

**Authors:** Eben Hillyer


### ARTICLE V.

*Extracts from the Records of the Atlanta Medical Society.* By EBEN HILLYER, M. D., Secretary. Feb. 28th, 1856.

*Rupture of the Uterus.*—Dr. W. F. Westmoreland reported the following:--He was called at 10 o'clock, P. M., Feb. 15th, to a colored woman in labor. Upon his arrival, he learned from the old midwife in attendance, that his patient was taken in labor at 10 o'clock the night previous, with severe labor pains, which continued, with great severity, about fourteen hours, when they became much less intense, and upon his arrival, had almost entirely subsided. He further learned, that this was the third labor. The woman was, apparently, considerably exausted; pulse 130. Upon examination, he found the os uteri sufficiently dilated, but the presenting part not yet engaged in the superior straight.

At his first examination, he did not satisfy himself as to the presentation, but subsequently, he determined that it was the neck and shoulder which was most prominent. After one or two ineffectual efforts to rectify the position of the child, he determined to turn and deliver, as the situation of his patient seemed to demand a hasty delivery. Upon introducing his hand, the presenting part receded without much force, and he very soon reached the left foot, which, after making several ineffectual efforts for the other, he brought down without difficulty. After delivering the leg to near the knee, he found it impossible to proceed further. After several attempts, he then tried to return the foot and leg, but found it impossible. Judging something unusual, he now attempted to make an examination, as well as he could do with the thigh of the child in the vagina, to learn, if possible, the cause of the difficulty. He detected something about the uterus unusual and peculiar, and for the first time, he suspected a laceration of its structure, but was not confident that it existed. He now dispatched a message for Dr. Boring, who arrived about ten o'clock, A. M. After Dr. B. had made several ineffectual efforts to deliver, they determined to amputate the thigh already delivered, which was done, and bring down the other foot, which was found in the abdominal cavity, behind the syphasis pubis. It was brought down, and the child delivered to the arms without much difficulty. The soft parts were by this time very much swolen, and quite tender. They did not deliver the arms until they had broke down the thorax. They found it impossible to deliver the head without breakiug it down, which was done by tearing away the inferior maxilary, the superior maxilary, and malar bones, and perforating the cranium through the base, which was rendered very tedious from the tumefaction of the parts. Introducing the hand, after the delivery of the child, Dr. W. found an extensive transverse rent in the uterus, in its anterior surface. The placenta was detached, and in the cavity of the peritoneum. The cause of the difficulty of delivery was obvious, viz: the child being out of the uterus.

The woman lived twenty-eight hours after the rupture, it occurring when the pains changed their character, which was at one o'clock, P. M. The Doctor was called at 10 o'clock, P. M. The patient lived three hours after delivery.

*Is Consumption infectious?*—Dr. W. F. Westmoreland mentioned the following as a singular coincidence:—He had under his charge a gentleman with tubercular phthisis, who, after lingering some months, died. In a few months his wife complained of a cough, which distressed her. Dr. W.'s fears were aroused; he made a physical examination, and was convinced that the lady had a deposit of tubercles. None of her family were ever known to have the disease. She was, at the time of her husband's death, a healthy woman, of full habit, and no visible cause for the accession of tuberculosis. She had nursed her husband through all his illness, and slept in the same room, and part of the time in the same bed. The progress of the disease was rapid, and fatal with her; in less than six months, she had followed her husband. Dr. Boring was of the opinion, that there was decidedly some grounds for considering tuberculosis infectious. He witnessed the cases reported by Dr. Westmoreland. He mentioned another case in which the husband died; his wife, a healthy woman, not of a consumptive family, nor physically liable to tuberculosis, was attacked, and died shortly after her husband. Another case in a family, none of whom were ever known to have Consumption, neither on the mother's or father's side. The eldest son was attacked, and died. Two months after, a younger sister, who was with him during his illness, showd symptoms of the disease, which were soon manifest; she, too, shortly died. Not long after, a still younger sister died as the elder one had. Last, a married sister, who was with the family during all the illness, was attacked, and died also.

Dr. B. remarked that there was a singular fatality in these cases to the persons connected with the invalids during their illness; and he was inclined to the opinion, the disease was infectious.

Dr. John G. Westmoreland was not prepared to say that tuberculosis was infectious, but that he had witnessed some similar coincidences. He had under his charge, a gentleman in the legal profession, who was attacked with clergyman's sore-throat, which resisted treatment; finally, there was a cough, then a rapid dissolution following a tubercular deposit. He died, and shortly after, a brother was taken and died in the same way. A young man living in the same house, no relation to them, was carried off in a similar manner: all being first attacked with the irritation in the throat.

Dr. Coe remarked that inflammation of a mucus membrane would often develop tubercles, when there was the tubercular habit.

Dr. Oliver mentioned a case in which the wife followed the husband soon after his death, and none of her family were ever known to have consumption.

[The contagious nature of tuberculosis is believed by many high in authority. This opinion is held extensively in the North of Europe, while in the South of Europe, its contagiousness is opposed. Morgagni is of opinion that it is contagious. In Italy, a consumptive is looked upon with as much fear as one with the plague. I do not believe that consumption is contagious. A diathasis is not communicable from one person to another. "Dr. Watson thinks that it cannot be communicated to a person of sound constitution." There are very few families who are entirely exempt from a taint of strumous cacexia in their pedigree, and it may have been dormant for several generations, and only waiting for the propitious moment to arrive to develop it. Such as the watching, want of rest, confinement in a sick room, and above all, intense mental anxiety, which is known to be one of the most certain causes to forward the hereditary predisposition of this disease. There is not sufficient testimony in favor of contagion: these cases are too isolated. Were this opinion correct, there would be many more instances in proof of it, for we rarely hear of the attendants of consumptives dying without we know them to be of consumptive family.—*Secretary.*]

*Case of Vesicular Eruption from the Administration of Iodide of Potassium.* Reported by DR. W. F. WESTMORELAND.—A negro woman was sent to Dr. W.'s Infirmary some four weeks ago, for treatment. From the following symptoms, it was evident she was suffering from tertiary syphilis: an offensive discharge from the nose; ulceration of the throat, extending to the larynx, producing almost complete aphonia, with some difficulty of respiration; an eruption upon the forehead, back, and breast, which was evidently syphilitic. She had been under treatment near a year, and for the past few months in charge of a "*Steamer.*" Dr. W.'s diagnosis was tertiary syphilis; he prescribed for her the iodide of potassium and the syrup of gentian, dissolving the salt in the syrup and giving from five to seven grs. at a dose morning, noon, and night. In thirty-six hours after the first dose, there appeared vessicles filled with serum, much resembling the vessicles produced by cantharides; they were in size from that of a half dime to that of a half dollar. Thinking this merely a coincidence, he continued the prescription, and in twelve hours the eruption had greatly increased. He now suspended the remedy, and in three or four days all had disappeared. There was some reaction during the eruption. At the expiration of a week, he again commenced to administer the same prescription, only giving about ten grs. in the entire twenty-four hours. The same result was presented a second time, but not until the third day after the prescription was resumed. The iodide of potassium was again discontinued, and the eruption disappeared in a few days. To satisfy himself thoroughly, he commenced its use a third time, giving only five or six grs. in the twenty-four hours, with still the same result.

She, each time, complained of a burning in the stomach and throat, before the appearance of the vessicles.

Large quantities of fluids were given during the administration of the medicine, in the two last experiments.

This was the first time Dr. Westmoreland had seen such a result from the use of this remedy, nor does he recolloct any like phenomena from its administration on record.

[That iodide of potassium produces peculiar effects upon the skin, has been observed by others. M. Ricord says, that it has peculiar effects upon some constitutions, such as various eruptions upon the skin, &c. Dr. A. Van Buren observes, that eruptions upon the skin was a very common effect of iodide potassium, given in long continued and large doses to patients in the Belvue Hospital.—*Secretary.*]

